# A Systematic Review on the Accuracy of Diagnostic Procedures for Infravesical Obstruction in Boys

**DOI:** 10.1371/journal.pone.0085474

**Published:** 2014-02-20

**Authors:** Pauline M. L. Hennus, Laetitia M. O. de Kort, J. L. H. Bosch, Tom P. V. M. de Jong, Geert J. M. G. van der Heijden

**Affiliations:** 1 Department of Urology, University Medical Center Utrecht, Utrecht, the Netherlands; 2 Pediatric Renal Center, Wilhelmina Children’s Hospital and University Medical Center Utrecht, Utrecht, the Netherlands; 3 Department of Epidemiology, Division Julius Center for Health Sciences and Primary Care, University Medical Center Utrecht, Utrecht, the Netherlands; 4 Department of Social Dentistry, Academic Centre for Dentistry Amsterdam (ACTA), University of Amsterdam and VU University Amsterdam, Amsterdam, the Netherlands; Centre for Inflammation Research, United Kingdom

## Abstract

**Background:**

Infravesical obstruction leads to kidney and bladder dysfunction in a significant proportion of boys. The aim of this review is to determine the value of diagnostic tests for ascertainment of infravesical obstruction in boys.

**Methodology:**

We searched PubMed and EMBASE databases until January 1, 2013, to identify papers that described original diagnostic accuracy research for infravesical obstruction in boys. We extracted information on (1) patient characteristics and clinical presentation of PUV and (2) diagnostic pathway, (3) diagnostic accuracy measures and (4) assessed risk of bias.

**Principal Findings:**

We retrieved 15 studies describing various diagnostic pathways in 1,189 boys suspected for infravesical obstruction. The included studies reflect a broad clinical spectrum of patients, but all failed to present a standardised approach to confirm the presence and severity of obstruction. The risk of bias of included studies is rather high due to work-up bias and missing data.

**Conclusions:**

As a consequence of low quality of methods of the available studies we put little confidence in the reported estimates for the diagnostic accuracy of US, VCUG and new additional tests for ruling in or ruling out infravesical obstruction. To date, firm evidence to support common diagnostic pathways is lacking. Hence, we are unable to draw conclusions on diagnostic accuracy of tests for infravesical obstruction. In order to be able to standardise the diagnostic pathway for infravesical obstruction, adequate design and transparent reporting is mandatory.

## Introduction

Infravesical obstruction, mostly caused by posterior urethral valves (PUV) occurrs in about 1 of 8000 pregnancies, leads to bladder dysfunction and forms the most common cause of chronic renal disease in a significant portion of boys. [1] Clinical presentations of infravesical obstruction range from late presentation by mild lower urinary tract symptoms (LUTS) and recurrent urinary tract infections (UTI) to early presentation with severe sequelae such as hydronephrosis with subsequent renal damage. [2], [3].

In some exceptional cases, history may strongly indicates infravesical obstruction, e.g. straining during voiding, making further diagnostic procedures superfluous. In most patients, however, adequate diagnostic tools are indispensable. At physiologic level, urethral obstruction should be defined as increased resistance to the flow of urine. [4] However, this is not feasible in for instance young boys who are not toilet trained.

Ultrasound (US) is widely used, especially to visualize the upper urinary tract. It is a non-invasive low cost test which carries no radiation exposure. Although US has shown to be sensitive for detecting hydronephrosis and a thickened bladder wall [5], infravesical obstruction is sometimes present in a milder form without these characteristics. [2], [3] Controversy remains concerning the role of relatively mild obstruction.

Many use voiding cystourethrogram (VCUG) to confirm findings from triage by US. Although VCUG is a useful tool for detecting vesicoureteral reflux, and examining the capacity and contour of the bladders; children are exposed to radiation and it is known that observer variability may reduce the accuracy of urethra assessment. [6], [7] Furthermore, the urethra can be clearly visualized using urethrocystoscopy (UCS). However, UCS is invasive and warrants general anaesthesia in paediatric patients and interobserver variability has been reported to reduce the accuracy of ascertainment of an obstruction. [8].

To date, new imaging modalities as voiding ultrasonography (VUS) and magnetic resonance urography (MRU) have been employed on a small scale to assess infravesical obstruction.

Several diagnostic tests and diagnostic pathways are used in clinical practice to diagnose infravesical obstruction. Despite the diagnostic possibilities outlined above the diagnostic value of the institutional tests and pathways has not been systematically evaluated and summarized. To compare two diagnostic tests, the difference in performance needs to be estimated against a common reference test. A variety of tests including urinary tract US, VCUG and UCS evaluation have been suggested as diagnostic measures for ascertainment of infravesical obstruction, either alone or as part of a diagnostic pathway. There is, however, no consensus about such a standardized reference test. Moreover, a definite diagnostic approach for infravesical obstruction in boys remains unclear.

To date, a standardized reference test for infravesical obstruction is lacking. Based on theory obstruction should be established by a pressure-flow study. But in daily practice UCS is used as the final in the series of investigations to be performed in the search of infravesical obstruction. However, one should realise that findings from all tests, including UCS, are based on subjective judgements. For this analysis we have chosen the findings during UCS as the primary endpoint because there is fair to good consensus among pediatric urologists on UCS as the reference standard for confirming the presence of infravesical obstruction. [8].

An accurate and reliable diagnostic approach of infravesical obstruction should promote timely and accurate diagnosis, reduce the use of unnecessary, expensive and invasive diagnostic tools, and should facilitate an early start of appropriate treatment.

Therefore, the aim of this review is to determine which clinical diagnostic test alone or in combination is most accurate and reliable in the diagnosis of infravesical obstruction in boys as confirmed with UCS.

## Patients and Methods

### Search Process

This study was conducted according to the PRISMA (Preferred reporting items for systematic reviews and meta-analyses) guidelines (Table S1– PRISMA 2009 checklist). [9] A literature search has been performed up to January 1^st^, 2013 in PubMed and EMBASE. Various synonyms were used for infravesical obstruction and endoscopic treatment and they were combined with synonyms for children (Table S2– Search strategy).

**Figure 1:**
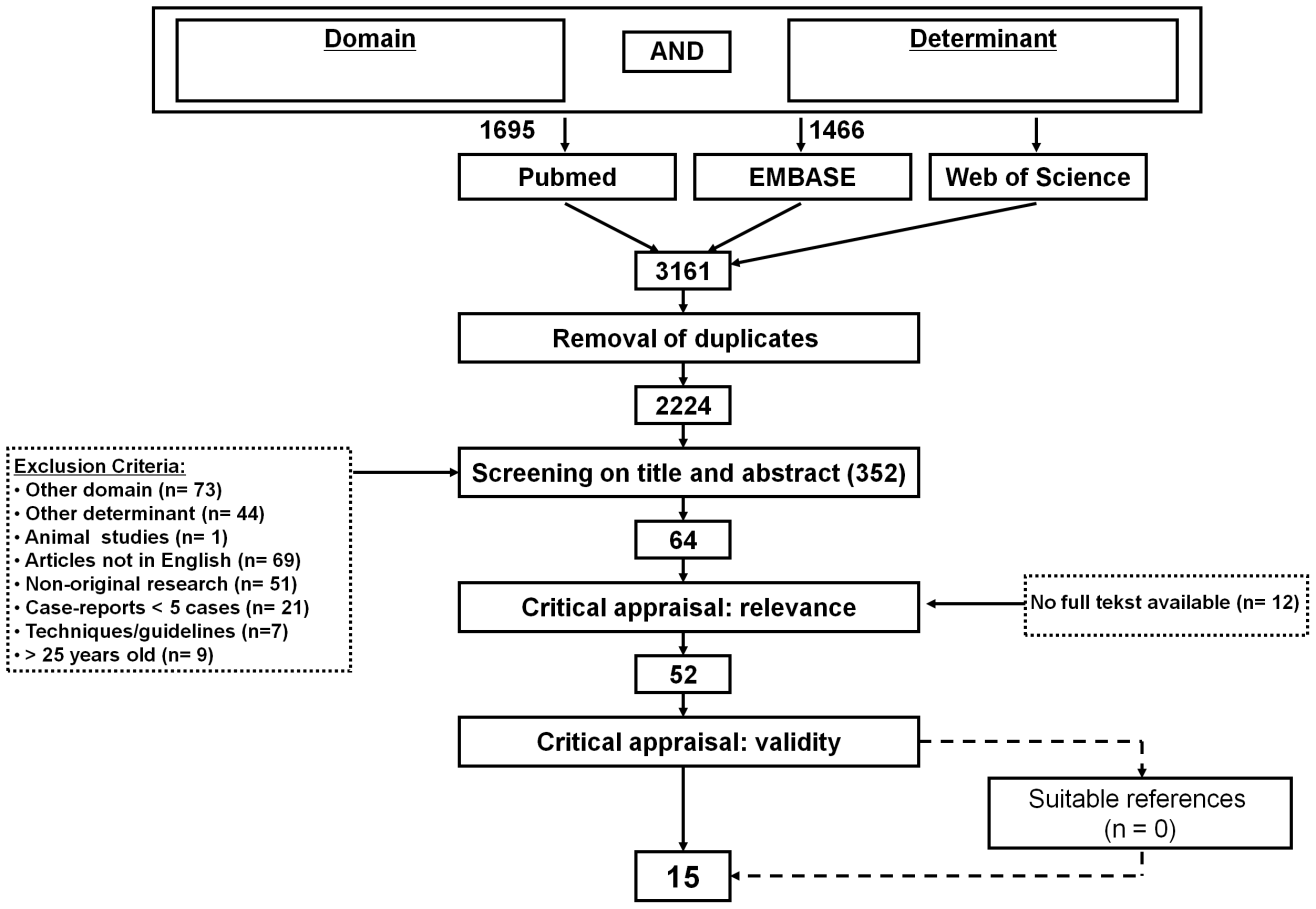
Flow chart.

Two investigators (PH and LdK) independently screened the titles and abstracts of all the retrieved articles. They selected studies reporting original data on diagnostic measurements in patients suspected of infravesical obstruction. They also excluded non-English language articles, studies with five or less children, review articles, animal studies and studies that enrolled only patients with confirmed diagnoses. To retrieve possibly omitted studies references of included and related articles have been checked. The complete flowchart is presented in Figure 1.

### Risk of bias assessment and principal endpoints

Assessment of study methods was conducted by two of the authors (PH, GvdH) using the QUADAS-2 tool, consisting of 4 domains: patient selection, index test, reference standard, and flow and timing, Table 1. [10] For the four QUADAS-2 domains we report for “patient selection” the relevant disease characteristics of patients included; for “index test” the number of patients in which the test was performed and the number of patients with a positive index test; for “reference standard” the number of patients in which an infravesical obstruction was verified and the number of confirmed cases; and for “flow and timing” we report the diagnostic pathway established by extracting the type and sequence of performed tests. In addition we indicate the risk of bias due to work-up and partial verification. Discrepancies between reviewers in selection and risk of bias assessment were resolved by discussion. Hence, presented results are based on full consensus. The endpoint for this review was the presence of urethral obstruction confirmed by UCS.

**Table 1:** Risk of bias assesment, Quadas-2 criteria.

	Methods criteria											
	Patient Selection				Index test(s)				Reference standard			
Study	1	2	3	4	5	6	7	8	9	10	11	12
1. Mate 2003	Yes	Yes	Yes	Yes	no	No	No	no	No	No	No	No
2. Duran 2009	Yes	Yes	Yes	Yes	no	No	No	no	No	No	No	No
3. Goldman 2000	yes	yes	yes	yes	yes	No	No	no	No	No	No	No
4. Oliveira 2000	Yes	Yes	Yes	No	yes	No	No	no	No	No	No	No
5. Ahmadzadeh 2007	yes	Yes	yes	No	yes	No	No	no	No	No	No	No
6. Berrocal 2005	Yes	Yes	yes	Yes	no	no	No	no	No	No	No	No
7. Bosio 2002	Yes	Yes	Yes	Yes	no	No	No	no	No	No	yes	yes
8. Kaefer 1997	Yes	Yes	Yes	No	yes	No	No	no	No	No	yes	yes
9. Nakamura 2010	Yes	Yes	Yes	Yes	yes	No	Yes	Yes	No	No	yes	yes
10. Kihara 2008	Yes	Yes	Yes	Yes	yes	No	Yes	Yes	No	No	Yes	Yes
11. De Kort 2003	Yes	Yes	Yes	Yes	yes	No	Yes	Yes	No	No	Yes	Yes
12. Chaumoitre 2004	Yes	Yes	Yes	No	yes	No	No	No	No	No	yes	yes
13. Cohen 1994	Yes	unclear	yes	yes	yes	No	No	no	No	No	yes	yes
14. Payabvash 2008	Yes	Yes	Yes	Yes	yes	No	No	No	Yes	unclear	Yes	Yes
15. De Kort 2004	Yes	Yes	Yes	Yes	yes	No	yes	yes	yes	yes	yes	yes

*Population*

1.a consecutive or random sample of patients were enrolledyesnounclear.

2.enrolment preceded verification of the disease status (case-control design was avoided) yesnounclear.

3.inappropriate exclusions were avoided yesnounclear.

4.all included patients were included in the data analysis yesnounclear.

*Index test(s)*

5.the index test was done in all patients yesnounclear.

6.the index test was interpreted without knowledge of the disease statusyesnounclear.

7.a threshold was used to interpret the index test yesnounclear.

8.the threshold was pre-specified yesnounclear.

*Reference standard*

9.the disease status was verified in all patients yesnounclear.

10.the disease status was verified without knowledge of the index test result yesnounclear.

11.the same reference standard was used in all patientsyesnounclear.

12.the reference standard allowed correct classification of the disease statusyesnounclear.

### Diagnostic pathways

**Figure 2:**
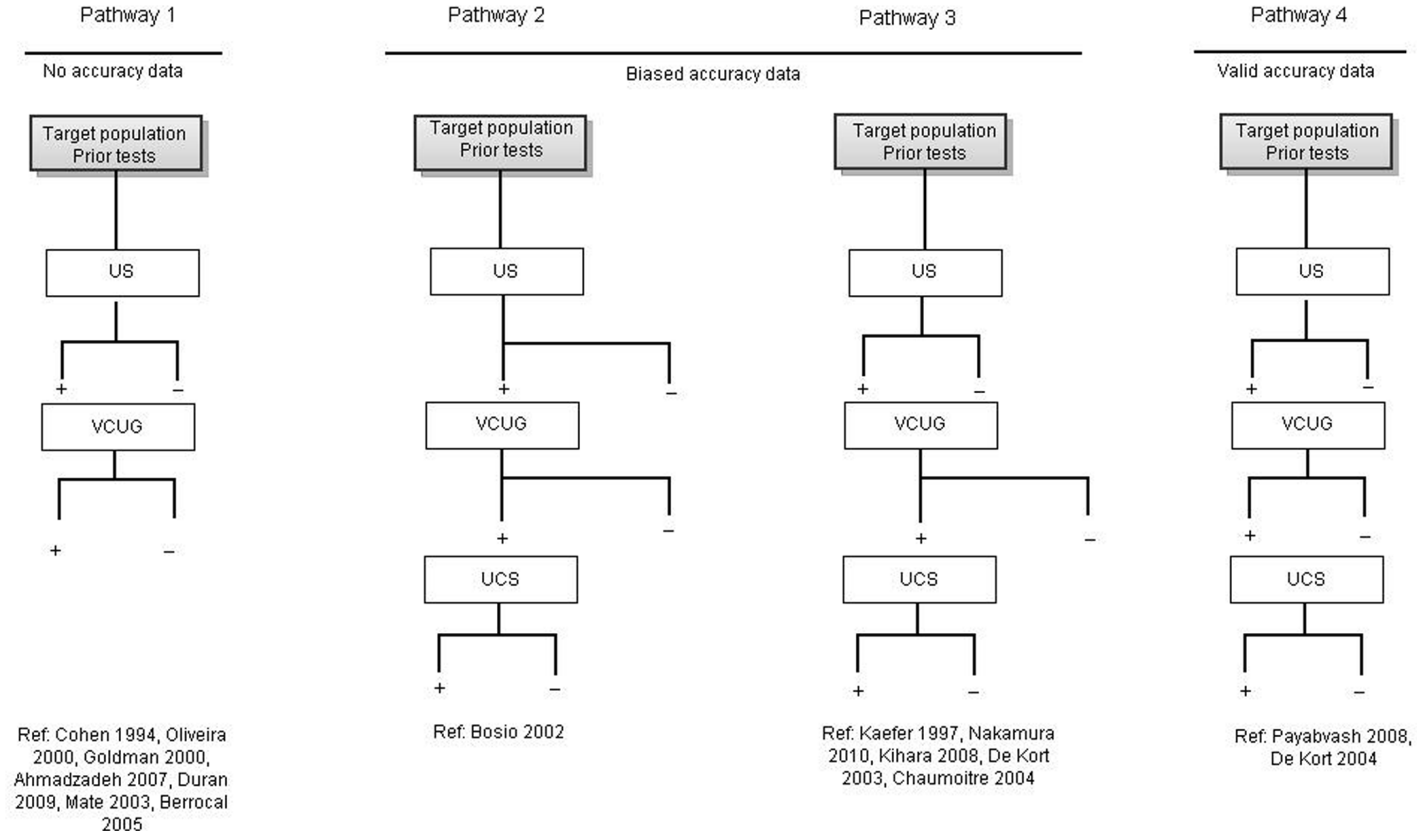
Diagnostic pathways. Legend: US = ultrasound, VCUG = voiding cystourethrogram, UCS = urethrocystoscopy. Pathway 1 performs US and VCUG in patients with positive US or in all included patients. Pathway 2 performs US in all, VCUG in patients with positive US and UCS in patients with positive VCUG. Pathway 3 perform US and VCUG in all patients, UCS is performed in patients with positive US and positive VCUG. Pathway 4 performs US, VCUG and UCS in all patients.

Different diagnostic pathways of established diagnostic modalities were used in diagnostic studies for infravesical obstruction. Because traditional pathways consist of several tests, four different pathways on the order, sequence and completeness of tests were defined to examine the added value of the different tests, see Figure 2.

In *pathway 1*, after US of the urinary tract, VCUG is performed in patients with a positive US or in all included patients. A reference test for verification of the endpoint however has not at all been performed. Therefore such study does not provide data that can serve to estimate the diagnostic accuracy of both US and VCUG.

In *pathway 2* US is performed in all patients; VCUG only in patients with a positive US. In addition, UCS is performed only in patients with positive VCUG. So, due to partial verification of the endpoint this pathway results in biased accuracy measures for both VCUG and US.

In *pathway 3* US and VCUG are performed in all patients, UCS is performed only in cases with a positive VCUG. So, due to partial verification of the endpoint this pathway results in biased accuracy measures for both VCUG and VUS.

In *pathway 4* US, VCUG and UCS are performed in all patients irrespective of other outcomes of the tests, and is therefore the optimal design for potentially providing unbiased diagnostic accuracy data.

## Results

### Study retrieval

We identified 3,161 titles, of which 3,109 studies did not meet the selection criteria or were duplicate publications retrieved from the different databases. After the review of 52 full-text studies, cross-reference checking did not reveal additional articles. Fifteen publications remained that met all inclusion criteria, and no articles were retrieved by cross-reference checking. Altogether, they included a total of 1,189 patients that met all inclusion criteria and were included for further assessment. Results of study retrieval are shown in Figure 1.

Assessment of study methods was conducted by two of the authors (PH, GvdH) using the QUADAS-2 tool, consisting of 4 domains: patient selection, index test, reference standard, and flow and timing, Table 1. [10] For the four QUADAS-2 domains we report for “patient selection” the relevant disease characteristics of patients included; for “index test” the number of patients in which the test was performed and the number of patients with a positive index test; for “reference standard” the number of patients in which an infravesical obstruction was verified and the number of confirmed cases; and for “flow and timing” we report the diagnostic pathway established by the extracting the type and sequence of performed tests. In addition we indicate the risk of bias due to work-up and partial verification.

### Quality assessment of diagnostic accuracy studies

Risk of bias of individual studies is summarized in Table 1. Domain “patient selection” carried low risk of bias, although due to missing data and loss to follow-up 4 studies did not include all patients in the data analysis. Domain “index test” carried high risk of bias; four studies did not perform the index text in all patients, the test was never performed without knowledge of the disease status and a (pre-specified) threshold was only clarified in four studies. Furthermore, domain “reference test” carried high risk of bias due to not performing the reference in all patients in 13 of the included studies. As can be seen in Table 1 and 2, domain “flow and timing” carries high risk of bias because VCUG and UCS are not performed in all included patients in 13 studies and the number of positive tests has not been reported for all individual tests in most studies.

**Table 2:** Synopsis included studies.

					Proportion positive of tests performed			
*Study*	*Pathway*	*Population*	*Sample size*	*Prevalence infr.obstr.*	*US*	*VCUG*	*UCS*	*Other*
**1. Mate 2003**	1	Case-mix	244	0.02 (4/244)	n/244	4/4		4/244 (VUS/TPUS) 1 ^,^ 5
**2. Duran 2009**	1	UTI/suspected for VUR	99	0.02 (2/99)	n/99	2/4		4/99 (VUS) 1
**3. Goldman 2000**	1	UTI <8 weeks	45	0.02 (1/45)	12/45	1/45		
**4. Oliveira 2000**	1	Hydronephrosis	103	0.14 (14/103)	n/103	14/103		
**5. Ahmadzadeh** **2007**	1	UTI	26	0.08 (2/26)	n/26	2/26		
**6. Berrocal 2005**	1	Case-mix suspected forVUR (56% after UTI)	87	0.03 (3/87)	n/87	3/87		3/87 (VUS)^1^
**7. Bosio 2002**	2	Hydronephrosis/UTI	100	0.08 (8/100)	n/100	8/8	8/8	n/100 (VCUS)^3^
**8. Kaefer 1997**	3	Hydronephrosis	15	0.53 (8/15)	n/15	8/15	8/8	
**9. Nakamura 2010**	3	Persistent nocturnalenuresis	43	0.47 (20/43)	n/43	22/43	20/22	n/22 (Uroflow, CMG, PFS)^2^
**10. Kihara 2008**	3	LUTS	37	0.24 (9/37)	n/37	17/37	9/17	
**11. De Kort 2003**	3	Case-mix	65	0.75 (49/65)	n/65	n/65*	49/56	n/65 UDO
**12. Chaumoitre** **2004**	3	Case-mix	123	0.02 (3/123)	n/123	3/123	3/s.c.^6^	
**13. Cohen 1994**	3	Hydronephrosis	10	0.50 (5/10)	n/10	5/10	4/4	5/10 (TPUS)^5^
**14. Payabvash 2008**	4	Unclear	61	0.28 (17/61)	n/61	17/61	17/61	13/61 (MRU)^4^
**15. De Kort 2004**	4	Case-mix	72	0.76 (55/72)	n/72	24/72	55/72	

1 VUS = voiding ultrasound,

2 CMG = cystometrogram, PFS = pressure flow study,

3 VCUS = voiding cysto urethrosonography,

4 MRU = magnetic resonance urography,

5 TPUS = transperineal ultrasound, s.c. = suspect cases, number unknown.

^*^VUDO was performed.

n = number of positive tests unknown.

### Initial presentation

The reported prevalence of infravesical obstruction varied widely between studies.

Except for one study [11], clinical presentation of the included patients was described in all articles, see Table 2. Although all patients were clinically suspected for infravesical obstruction, initial presentation differed between studies. Patients with infravesical obstruction, in general presented with hydronephrosis, UTI, urinary incontinence (UI) or LUTS. Three studies only including patients with hydronephrosis report PUV in 27 (21%) out of 128 patients, (range 14–53%).[12]–[14] Two studies including patients presenting with UTI alone reported PUV in 3 (4%) out of 71 patients, (range 2–8%). Three studies reporting on patients with UI or LUTS reported infravesical obstruction in 78 (54%) out of 145 patients, (range 24–75%).[15]–[17] The other six studies included a heterogeneous patient group (case-mix) presenting with hydronephrosis, UTI, UI and/or LUTS reportedinfravesical obstruction in 75 of 725 patients, 10% (range 2–76%).[6], [18]–[22] There appear to be more patients with an infravesical obstruction in patients presenting with hydronephrosis, UI and LUTS.

### Diagnostic pathways (Figure 2)

The included studies reported different tests and different pathways to diagnose infravesical obstruction. In most included studies all subjects underwent the index test, but only in patients with a positive index test the disease presence was verified using a reference standard.

All 15 included studies performed US first in all patients. Twelve studies performed VCUG after US in all patients. Three studies performed VCUG only in suspected patients. [19], [21], [22] Two studies performed UCS in all patients [6], [11] and seven studies performed UCS in patients suspected for infravesical obstruction based on a positive VCUG.[13], [15]–[17], [20], [23], [24] In the remaining six studies UCS was not used to verify infravesical obstruction. In three of these studies, a head to head comparison of VUS with VCUG was performed. As shown in Figure 2, six studies followed *pathway 1*, one study *pathway 2*, six studies *pathway 3* and two studies *pathway 4*.

#### Pathway 1

Six studies were classified as *pathway 1* and reported the use of both postnatal US and VCUG in all patients suspected for infravesical obstruction. One study included patients who presented with hydronephrosis, three studied included patients presenting with UTI and two included a case-mix, Table 2. These studies did not provide any data on the diagnostic accuracy of either diagnostic modality, because UCS has not been performed to confirm infravesical obstruction. VCUG was only performed in patients with positive VUS, for confirmation of infravesical obstruction in all patients suspected for obstruction. [13], [18] The best these studies provide is full agreement for the positivity for both VUS and VCUG, 100%. There is bias in these diagnostic studies due to the lack of endpoint verification. Still, the seven studies following pathway 1 claimed to have infravesical obstruction confirmed in 26 (4%) out of 604 boys, (ranging 2%–14%). [13], [14], [18], [22], [25], [26] They considered infravesical obstruction (PUV in most patients) confirmed by VCUG. Three of these studies performed VUS as a replacement test [27] in all patients alongside VCUG as reported reference test. Two studies verified all PUV both in VUS and VCUG [18], [22], one study and one study verified PUV in 2 (50%) out of 4 patients suspected for PUV. [21].

#### Pathway 2

One study was classified as *pathway 2* including patients presenting with hydronephrosis and UTI. [23] Investigators performed US and VCUS as triage test in all 100 patients [19], [21], [22] VCUG was performed in 8 patients suspected of infravesical obstruction based on US and VCUS both being positive, followed by UCS in 8 cases based on a positive VCUG confirming infravesical obstruction in all 8 suspect cases (8%). There was considerable uncertainty about the point estimates derived from this study. The risk of bias is considerable due to partial verification in this pathway for a substantial part of the study population (92%).

#### Pathway 3

Six studies were classified as *pathway 3*. One study only included patients with persistent nocturnal enuresis, another study included patients with LUTS and two studies included a case-mix, Table 2. All five studies performed postnatal US and VCUG in all patients. For confirmation of obstruction UCS, was only performed when US and VCUG both were positive. [12], [15]–[17], [20]An infravesical obstruction was found in 84 (29%) out of 293 (range 2–75%). There is bias due to partial verification of the endpoint of disease.

#### Pathway 4

Two studies were classified as *pathway 4* and performed US, VCUG and UCS in all patients suspected for infravesical obstruction. [6], [11] Both studies included a case-mix. Infravesical obstruction was diagnosed in 72 (44%) out of 162 boys (range 28–76%).

In the study by Payabvash et al. [11] MRU, US, VCUG and UCS were performed in all patients. UCS was used as reference test and confirmed all 17 cases with PUV suspected based on VCUG. Unfortunately, only the prevalence (0.28; n/17) could be calculated, but the reported data did not allow to calculate PPV, NPV and added value of VCUG or US. Sufficient conclusive clues from MRU images resulted in the the diagnosis of infravesical obstruction for 13 out of 17 patients. Postnatal US detected 8 out of 17 PUV, 47%. Also, in this study, MRU was performed as add-on test in those children with insufficient conventional imaging, when a diagnosis could not be established or if a co-existing urogenital anomaly was suspected. With UCS as reference standard, reported sensitivity for PUV was 76% for MRU. [11].

De Kort et al. [6] reported a prevalence of 0.76 of infravesical obstruction. Studying the raw patient data of de Kort et al., using UCS as reference test versus VCUG, the added value of VCUG when the test was positive was 0.07 so the risk of disease, i.e. obstruction, increased from 0.76 to a positive predictive value 0.83 presence when VCUG was positive. The added value of VCUG when the test was negative was 0.03, so the risk of not having disease/obstruction in the study population increased from 0.24 to a negative predictive value (NPV) of 0.27 presence when VCUG was negative. [6].

## Discussion

Of the 15 included studies, seven of the studies followed pathway 1. Due to the major shortcomings in the design of pathway 1, results cannot be used to provide conclusions. Also the six studies following both pathway 2 and pathway 3 provide biased data due to partial verification of the endpoint of disease. Of the two studies following pathway 4, only one study provided accurate data after retrieval of raw study data. There are too few data to draw firm conclusions. Furthermore, although one of the included studies performed pressure flow study and urodynamic study in boys with persistent incontinence, no clear data were reported on the performance as a diagnostic tool for infravesical obstruction. [15] Measurements were rather used to compare outcomes before and after transurethral treatment of urethral obstruction. Increased Qmax and increased bladder capacity were found after transurethral treatment.

For almost all included studies the risk of bias was rather high due to partial verification of the endpoint, lack of blinding of tests and absence of independent interpretation of their results. Moreover, selection and description of participants was reported insufficiently. Therefore, the best available study thus far provides limited evidence for the added value on positive or negative VCUG in ruling in or ruling out infravesical obstruction.

Most studies report biased outcomes for diagnostic accuracy. We showed that different diagnostic pathways are used to diagnose infravesical obstruction and divided these into four pathways. US was always performed in all patients. Some studies also performed VCUG in all cases, while some only performed VCUG in suspected cases after a positive US as a triage test. Half of the studies report verification of infravesical obstruction by UCS and only two studies report that UCS was performed in all patients. No association between clinical presentation and the followed pathway was found. This is probably due to the fact that the choice of the diagnostic pathway may depend on local customs and protocols and was not affected by the specific presenting symptomatology.

The prevalence of infravesical obstruction, mostly PUV, varied widely between studies. This can be affected by clinical and methodological factors. [28], [29] Notwithstanding all drawbacks, flaws and bias, infravesical obstruction was most frequently found in patients with hydronephrosis, mostly at young age, and in studies presenting patients with urinary incontinence or LUTS. Infravesical obstructions were less often reported in patients who presented with UTI. The higher prevalence of PUV in patients initially presenting with hydronephrosis might be explained by a relative overrepresentation of the more severe end of the spectrum. The high prevalence of infravesical obstruction found in two rather small studies of boys who initially presented with persistent nocturnal enuresis and LUTS might be due to long existent relatively mild obstruction. The other half of the included studies reported outcomes for a patient-mix, resulting in presence of an infravesical obstruction in 1 out of 10 patients.

Besides flaws and bias in almost all included studies, interobserver variability should be taken into account. Due to difficulties in the judgement of infravesical obstruction De Kort et al. described that observer variability played an important role in VCUG for the assessment of the urethra and it also played a role in UCS where judgment of the presence of urethral obstruction is up to the endoscopist performing the procedure. [6] However, although the assessment of the urethra by UCS is a subjective judgement, there is fair to good consensus among pediatric urologists. [8] Hence, results of the same diagnostic test may vary within and between different studies.

To our knowledge this is the first systematic review on the diagnostic methods for infravesical obstruction in boys. However, in interpreting our findings, some aspects need further consideration.

A set of minimal reporting standards for diagnostic research has been proposed and used in this review: QUADAS-2, a revised tool for the quality assessment of diagnostic accuracy studies. [10] Patient flow and work-up bias, described by the different pathways, remain a difficult reporting problem. From a clinical point of view it is understandable that not all patients underwent all tests, but for research all information is needed. Otherwise biased estimates might be found. The question remains, what the ideal pathway to diagnose infravesical obstruction might be. Unnecessary or excessive examinations may expose patients to risk without benefit, as radiation exposure in VCUG and anaesthesia when performing UCS in children. Without question US of the upper urinary tract is the first step in all patients suspected for infravesical obstruction. Therefore, US can be used as a triage test, an initial step in a diagnostic pathway to identify the group of patients who need further assessment. A triage test does not aim to improve the diagnostic accuracy of the existing test, but rather to reduce the number of individuals having an unnecessary more invasive test. However, an infravesical obstruction might still be present despite a normal US.

Furthermore, there is no consensus for a reference standard. To compare two diagnostic tests, the difference in accuracy needs to be estimated against a reference standard. This was not done for most studies. Two studies tested the accuracy of VCUG against UCS in all patients, only one provided useful information. Uncertainty remains whether VCUG and UCS should be performed in all patients or in selected cases. A study where all boys with clinical suspicion of infravesical obstruction go through a complete diagnostic work-up including US and VCUG in combination with pressure flow study will be necessary to avoid most forms of bias. Because of the lack of a standardized reference test, and despite the fact that obstruction – per definition - should be established by a pressure-flow study, we have chosen findings during UCS as the primary endpoint. UCS is the final in the series of investigations to be performed in the search of infravesical obstruction. Also, the majority of the boys included in the studies represent the severe end of the clinical spectrum. For this reason, the results may not be applicable for all patients.

Finally, shortcomings in the design of study may bias estimates of diagnostic accuracy, but the magnitude of the bias may vary from one situation to another. [28] Due to both the poor reporting and the identified sources of bias, invalid estimates of the diagnostic accuracy may result in claims about the appropriateness and clinical value eventually will lead to inadequate diagnosis of patients. Therefore, the marked variation in estimates should make clinicians cautious when reading studies reporting on the diagnostic accuracy of tests. It is important that such studies are properly designed and reported. Furthermore, it is striking that only one study performed pressure flow study. At physiologic level, urethral obstruction should be defined as increased resistance to the flow of urine. [4] To prove obstruction, intraluminal pressures need to be measured. However, determining pressures in the urinary tract may be technically challenging, especially in young children, and will generally require an invasive procedure. Moreover, the influence of a transurethral catheter during the procedure is unsure and might lead to an overestimation of the actual obstruction. Instead clinicians often rely on diagnostic modalities that measure a derivative value from of obstruction. These (indirect) measures of obstruction include conventional US, VCUG and UCS.

## Conclusion

As a consequence of low quality of methods of the available studies we put little confidence in the reported estimates for the diagnostic accuracy of US, VCUG and new additional tests for ruling in or ruling out infravesical obstruction. To date, firm evidence to support common diagnostic pathways is lacking. Hence, we are unable to draw conclusions on diagnostic accuracy of tests for infravesical obstruction. In order to be able to standardise the diagnostic pathway for infravesical obstruction, adequate design and transparent reporting is mandatory.

## Supporting Information

Table S1 **PRISMA 2009 Checklist.**
(DOC)

Table S2 **Search Strategy.**
(DOC)
