# Supplementary material for: A Systematic Review on the Accuracy of Diagnostic Procedures for Infravesical Obstruction in Boys
Source: PLoS One. 2014 Feb 20;9(2):e85474. doi: 10.1371/journal.pone.0085474 (PMC3930523; doi:10.1371/journal.pone.0085474)
Supplement: Table S2 — Search Strategy. (DOC) [file pone.0085474.s002.doc]

**Table S2. Search Strategy**

*Domain:*

(obstruction[Title/Abstract] OR obstructions[Title/Abstract] OR obstruent[Title/Abstract] OR obstructive[Title/Abstract] OR obstructed[Title/Abstract] OR stricture[Title/Abstract] AND infravesical[Title/Abstract] OR urethra[Title/Abstract] OR urethral[Title/Abstract] OR intraurethral[Title/Abstract] OR "urinary tract"[Title/Abstract] OR "urinary tracts"[Title/Abstract]) ***OR*** (urethra[Title/Abstract] OR urethral[Title/Abstract] OR flap[Title/Abstract] OR infravesical[Title/Abstract] AND (valve[Title/Abstract] OR valves[Title/Abstract]) ***OR*** (PUV[Title/Abstract] OR mohrmann[Title/Abstract] OR "cobb's collar"[Title/Abstract] OR diverticulum[Title/Abstract] OR "bladder neck"[Title/Abstract] OR syringocele[Title/Abstract] OR "meatal stenosis"[Title/Abstract] OR "navicular fossa"[Title/Abstract])

*Determinant:*

"voiding cystourethrogram"[Title/Abstract] OR VCUG[Title/Abstract] OR "voiding cystourethrography"[Title/Abstract] OR VCUS[Title/Abstract] OR urethrography[Title/Abstract] OR radiographic[Title/Abstract] OR radiological[Title/Abstract] OR imaging[Title/Abstract] OR DMSA[Title/Abstract] OR "dimercaptosuccinic acid"[Title/Abstract] OR urosonography[Title/Abstract] OR cystoscopy[Title/Abstract] OR cystourethroscopy[Title/Abstract] OR urethroscopy[Title/Abstract] OR ultrasound[Title/Abstract] OR ultrasonography[Title/Abstract] OR sonography[Title/Abstract] OR renography[Title/Abstract] OR urodynamic[Title/Abstract] OR urodynamics[Title/Abstract] OR "pressure flow study"[Title/Abstract] OR cystometry[Title/Abstract] OR diagnostic[Title/Abstract] OR diagnostics[Title/Abstract] OR diagnosis[Title/Abstract] OR diagnose[Title/Abstract] OR diagnosed[Title/Abstract] OR oxybutynin[Title/Abstract] OR uroflow[Title/Abstract] OR uroflowmetry[Title/Abstract] OR fluoroscopy[Title/Abstract]

*Children:*

child[Title/Abstract] OR children[Title/Abstract] OR boy[Title/Abstract] OR boys[Title/Abstract] OR pediatrics[Title/Abstract] OR pediatric[Title/Abstract] OR paediatrics[Title/Abstract] OR peadiatric[Title/Abstract] OR juvenile[Title/Abstract]

**SEARCH: Domain AND Determinant AND Children**.
